# Complete Genome Sequences of Two Rat Pegivirus Strains in Indonesia

**DOI:** 10.1128/MRA.00049-21

**Published:** 2021-03-18

**Authors:** Tsutomu Nishizawa, Mulyanto, Yumi Hatano, Kazumoto Murata, Hiroaki Okamoto

**Affiliations:** a Division of Virology, Department of Infection and Immunity, Jichi Medical University School of Medicine, Shimotsuke, Tochigi, Japan; b West Nusa Tenggara Hepatitis Laboratory, Mataram, Indonesia; c Immunobiology Laboratory, Faculty of Medicine, University of Mataram, Mataram, Indonesia; d Sakakibara Heart Institute Clinic, Shinjuku-ku, Tokyo, Japan; KU Leuven

## Abstract

The entire genome sequences of two pegivirus strains recovered from serum samples of wild rats (Rattus rattus) in Indonesia were determined. They possessed 11,013 to 11,014 nucleotides and differed from the reported rodent pegivirus strains within the *Pegivirus J* species of the genus *Pegivirus* by 12.7% to 40.9% in the near-entire coding region sequences.

## Abstract (precis)

The entire genome sequences of two pegivirus strains recovered from serum samples of wild rats (Rattus rattus) in Indonesia were determined. They possessed 11,013 to 11,014 nucleotides and differed from the reported rodent pegivirus strains within the *Pegivirus* J species of the genus *Pegivirus* by 12.7% to 40.9% in the near-entire coding region sequences.

## ANNOUNCEMENT

Pegiviruses belong to the genus *Pegivirus* within the family *Flaviviridae*. They infect various mammalian hosts (1), including humans, nonhuman primates, bats, horses, rodents, pigs (2, 3), and dolphins (4), and are classified into 11 species, *A* to *K* (2), and 1 proposed species, *L* (4).

**FIG 1:**
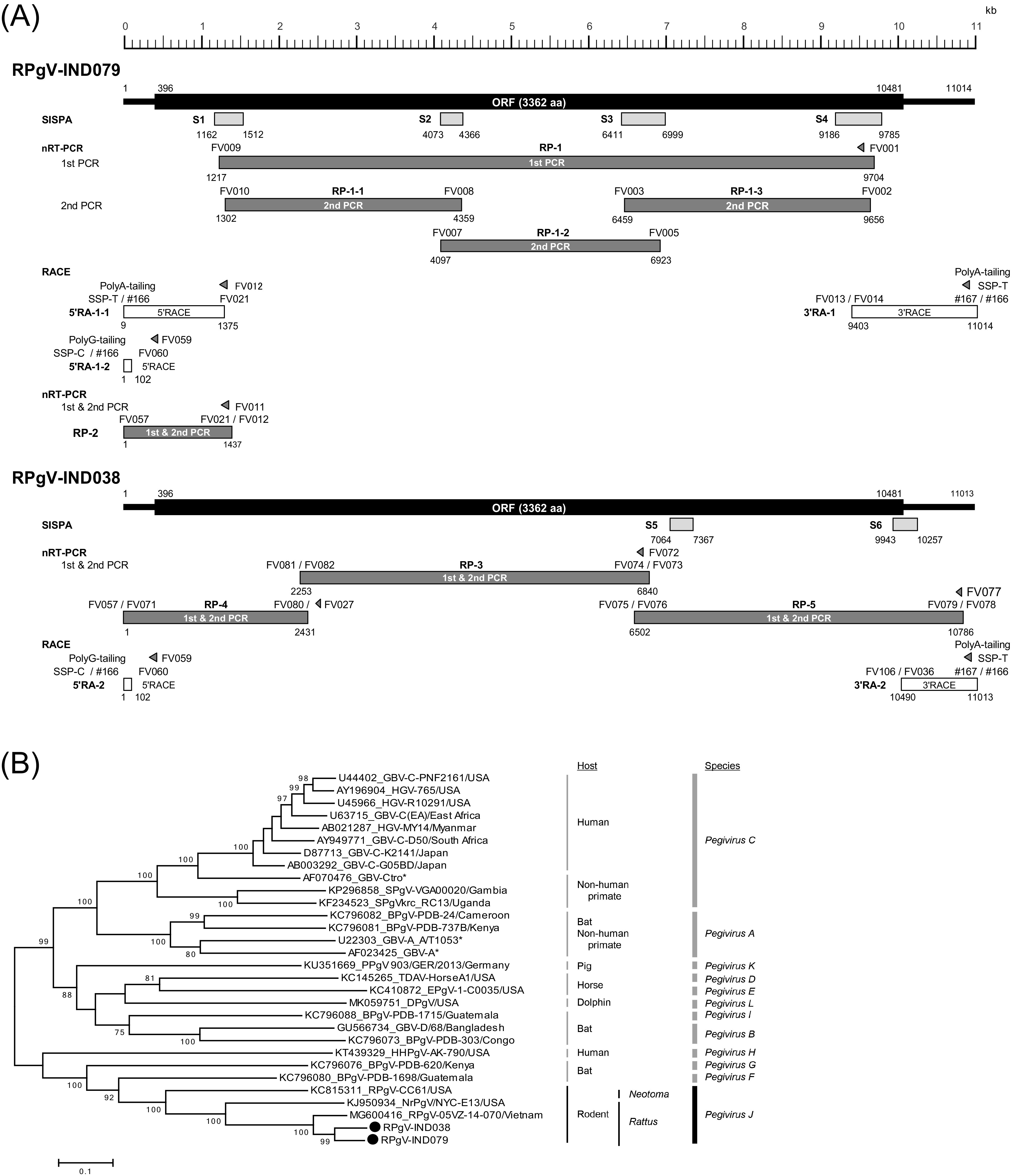
(A) Strategies for determining the complete genome sequences of the two rat pegivirus strains (RPgV-IND079 and RPgV-IND038) obtained in the present study. The light-gray boxes (S1 to S6) with nucleotide positions at both ends below the schematic organization of the rat pegivirus genome indicate the genomic areas amplified using the SISPA method (6), which includes reverse transcription using a random primer tagged with a known sequence, FR20RV-N6 (5′-GCCGGAGCTCTGCAGATATCNNNNNN-3′) with Superscript III (Thermo Fisher Scientific, Inc.) followed by amplification with *Ex Taq* polymerase (TaKaRa Bio, Inc., Shiga, Japan) using a primer with the underlined sequence of FR20RV-N6 (FR20RV, 5′-GCCGGAGCTCTGCAGATATC-3′). The dark-gray boxes (RP-1 to RP-5 and RP-1-1 to RP-1-3) with nucleotide positions at both ends depict the genomic areas amplified by nRT-PCR with primers (Table 1) and *Ex Taq* polymerase following reverse transcription with primers (Table 1) highlighted with triangles and Superscript III. Open boxes (5′RA-1-1, 5′RA-1-2, 5′RA-2, 3′RA-1, and 3′RA-2) with nucleotide positions at both ends indicate the genomic areas amplified by the 5′-RACE and 3′-RACE methods (7, 8). (Top) From the IND079 serum, four DNA fragments (S1 to S4) with nucleotide sequences similar to those of a rat pegivirus genome (DDBJ accession number MT085182) were obtained using the SISPA method. Using four pairs of primers (one pair for first-round PCR and three pairs for second-round PCR) generated based on the SISPA-derived sequences (Table 1), one DNA fragment (RP-1) and three DNA fragments (RP-1-1 to RP-1-3) were amplified by first-round PCR and second-round PCR, respectively, with *Ex Taq* polymerase following reverse transcription with Superscript III. The sequences of the 5′- and 3′-terminal regions (5′RA-1-1, 5′RA-1-2, and 3′RA-1) were amplified by the 5′-RACE and 3′-RACE methods with the primers synthesized based on the RP-1-1 or RP-1-3 sequences, respectively. The RP-2 fragment was amplified to confirm the 5′-terminal region sequence (nt 23 to 1355) since it was determined based on two consecutive RACE reactions. (Bottom) From the IND038 serum, two DNA fragments (S5 and S6) with nucleotide sequences similar to those of a rat pegivirus genome (MT085182) were obtained using the SISPA method. Using three pairs of primers (Table 1), three DNA fragments (RP-3 to RP-5) were amplified by nRT-PCR as described above. The 5′-RACE (5′RA-2) and 3′-RACE (3′RA-2) products were amplified with primers (Table 1) generated based on the RPgV-IND079 sequence. In the present study, all amplification products were sequenced two or more times on both strands directly or after cloning into pMD20 T-Vector (TaKaRa Bio, Inc.) (with the bidirectional primer-walking method, when needed) using an Applied Biosystems 3130xl genetic analyzer (Thermo Fisher Scientific, Inc.) with a BigDye Terminator v3.1 cycle sequencing kit (Thermo Fisher Scientific, Inc.). All reads were assembled using the Genetyx software program v13.0.1 (Genetyx Corp., Tokyo, Japan) to determine the complete genome sequences. (B) A phylogenetic tree of the nucleotide sequences of the entire coding region of the two strains (RPgV-IND038 and RPgV-IND079, highlighted with filled circles) obtained in the present study with 28 reported reference isolates of *Pegivirus A* to *K* and *Pegivirus L* proposed by Smith et al. (3) and Rodrigues et al. (4), respectively. The tree was constructed using the maximum likelihood method with the MEGA7 software program v7.02.26 (11) after alignment using the MUSCLE software program v3.5 (12). Each reference sequence is shown with the accession number, the strain name, and the name of the country. The bootstrap values (≥70%) for the nodes are indicated as a percentage of the data obtained from resampling 1,000 times. The scale bar represents the number of nucleotide substitutions per site. Asterisks indicate that countries where nonhuman primates were captured are not specified.

While RNA extracted from sera of five wild rats (Rattus rattus) in Indonesia (5) with TRIzol-LS (Thermo Fisher Scientific, Inc., Waltham, MA) was subjected to sequence-independent, single-primer amplification (SISPA) (6), 4 and 2 DNA fragments 84% to 91% identical to a rat pegivirus strain (DDBJ accession number MT085182) (NCBI BLAST search) covering 16.3% and 5.5% of the pegivirus genome were obtained from 2 rats, IND079 and IND038, respectively (Fig. 1A). From the IND079 serum, three partially overlapping DNA fragments were amplified by nested reverse transcription-PCR (nRT-PCR) using four primer pairs generated based on the SISPA-derived sequences (Table 1), and the near-entire coding region sequence of the RPgV-IND079 genome was determined. Nucleotide sequencing of both the 5′- and 3′-terminal regions was carried out using the rapid amplification of cDNA ends (RACE) method (Fig. 1A, top) using homopolymer tailing of dATP or dGTP with terminal deoxytidyl transferase and ATP with poly(A) polymerase (New England Biolabs, Ipswich, MA), respectively (7, 8). To confirm the 5′-terminal sequence, additional seminested RT-PCR (RP-2; Fig. 1A, top) was performed and subjected to sequence analyses. From the IND038 serum, three partially overlapping DNA fragments covering the entire coding region were amplified by nRT-PCR using three primer pairs generated based on well-conserved sequences of the rat pegivirus genomes, including the RPgV-IND079 genome (Table 1), and the entire coding region sequence of the RPgV-IND038 genome was determined. In addition, the 5′- and 3′-terminal region sequences were determined using the RACE method, as described above (Fig. 1A, bottom). In this study, each amplicon was sequenced two or more times using the Sanger method (see Fig. 1 legend for details).

**TABLE 1:** Primers used for the determination of the complete genome sequences of two rat pegiviruses, RPgV-IND079 and RPgV-IND038, in the present study^*a*^

Amplification	Strain	Name	Sequence (5′ to 3′)	Polarity	Position^*b*^	Region^*c*^ (reaction)
nRT-PCR	RPgV-IND079	FV001	GCCGTGGATCAGGAAGCTG	−	9686−9704	RP-1 (RT and 1st round PCR)
		FV009	TGGCTGTTGGACGAGCATTG	+	1217−1236	RP-1 (1st round PCR)
		FV008	GAAAAGTGGCGTAGAGCACG	−	4340−4359	RP-1-1 (2nd round PCR)
		FV010	TTCGGATTCATTGGCTGGGC	+	1302−1321	RP-1-1 (2nd round PCR)
		FV005	GGACAACAAGCGGTTCATCC	−	6904−6923	RP-1-2 (2nd round PCR)
		FV007	TTATGGTTCGGAACCCGTGG	+	4097−4116	RP-1-2 (2nd round PCR)
		FV002	CGCCGAAACTTTGAGGTAGC	−	9637−9656	RP-1-3 (2nd round PCR)
		FV003	TTGGAGACTCACCTAACTGC	+	6459−6478	RP-1-3 (2nd round PCR)
		FV011	TCCAGCCAGGAGCTGTAAGC	−	1443−1462	RP-2 (RT)
		FV012	GGCAGGACGTATTGCAGATG	−	1418−1437	RP-2 (1st round PCR)
		FV057	GGACTTCGGTCCCTCACCTAAC	+	1−22	RP-2 (1st and 2nd round PCR)
		FV021	GCCAAGCCTGCAGCATAGTG	−	1356−1375	RP-2 (2nd round PCR)
	RPgV-IND038	FV072	GCCATSGCTATSCCGGCAAC	−	6843−6862	RP-3 (RT)
		FV073	CAGCTCCCGGRCATAGRAGG	−	6821−6840	RP-3 (1st round PCR)
		FV081	GGCTTTGGTTGTTCGTCGAC	+	2253−2272	RP-3 (1st round PCR)
		FV074	CCCGTCAAGCARGGCAARGC	−	6801−6820	RP-3 (2nd round PCR)
		FV082	CCACGGTCTCATCAACTGTTGG	+	2273−2294	RP-3 (2nd round PCR)
		FV027	AGGCGTGAGCGCTTGTACTG	−	2412−2431	RP-4 (RT and 1st round PCR)
		FV057	GGACTTCGGTCCCTCACCTAAC	+	1−22	RP-4 (1st round PCR)
		FV080	CCACAACCAGTTGCTGGAGC	−	2350−2369	RP-4 (2nd round PCR)
		FV071	CAGTCAGCCACGACTGGCG	+	23−41	RP-4 (2nd round PCR)
		FV077	GCCTTACGGCCCCTTCGTG	−	10799−10817	RP-5 (RT)
		FV078	TCTGGCGCCGATCTACTGTC	−	10768−10787	RP-5 (1st round PCR)
		FV075	TGGGCTCAGCGCGGTCATG	+	6502−6520	RP-5 (1st round PCR)
		FV079	TACAGGCTGCGAGTCGCTTC	−	10735−10754	RP-5 (2nd round PCR)
		FV076	CGCTATCCTCAGTAGCGTCG	+	6530−6549	RP-5 (2nd round PCR)
5′ RACE	RPgV-IND079	FV012	GGCAGGACGTATTGCAGATG	−	1418−1437	5′RA-1-1 (RT and 1st round PCR)
		FV021	GCCAAGCCTGCAGCATAGTG	−	1356−1375	5′RA-1-1 (2nd round PCR)
	RPgV-IND079 and RPgV-IND038	FV059	CGCGTGAGCAGCCTATTCG	−	98−116	5′RA-1-2 (RT and 1st round PCR)
						5′RA-2 (RT and 1st round PCR)
		FV060	ATTCGCGCGCCTTACTAACG	−	83−102	5′RA-1-2 (2nd round PCR)
						5′RA-2 (2nd round PCR)
3′ RACE	RPgV-IND079 and RPgV-IND038	FV013	CACCAGTGTGCTACACGGTC	+	9403−9422	3′RA-1 (1st round PCR)
		FV014	CGCGAAGGCGTCGAATGAC	+	9485−9503	3′RA-1 (2nd round PCR)
		FV106	CTCAGGGCAGGAGGCTTAGG	+	10490−10509	3′RA-2 (1st round PCR)
		FV036	GAATAACCCCAGTCACGAAGG	+	10786−10806	3′RA-2 (2nd round PCR)
5′ RACE and 3′ RACE	RPgV-IND079 and RPgV-IND038	SSP-T^*d*^	AAGGATCCGTCGACATCGATAATACGTTTTTTTTTTTTTTT		NA	5′RA-1-1 (1st round PCR) 3′RA-1 and 3′RA-2 (RT)
		SSP-C^*d*^	AAGGATCCGTCGACATCGATAATACGCCCCCCCCCCCCCCC		NA	5′RA-1-2 and 5′RA-2 (1st round PCR)
		No. 166^*d*^	AAGGATCCGTCGACATCGAT		NA	5′RA-1-1 and 5′RA-1-2 (2nd round PCR) 5′RA-2 (2nd round PCR) 3′RA-1 and 3′RA-2 (1st round PCR)
		No. 167^*d*^	CCGTCGACATCGATAATACG		NA	3′RA-1 and 3′RA-2 (2nd round PCR)

a RT, reverse transcription; NA, not applicable.

b Nucleotide position in accordance with the genome sequence of RPgV-IND079.

c See Fig. 1.

d See reference 8.

The RPgV-IND038 and RPgV-IND079 genomes were 11,013 and 11,014 nucleotides (nt) long (G+C content, 62.0% to 62.1%) and possessed a long open reading frame of 10,086 nt (3,362 amino acids) with a 5′ untranslated region (UTR) of 395 nt and a 3′ UTR of 529 to 530 nt. They shared 90.7% identity over the entire genome.

A phylogenetic analysis conducted based on the entire coding region sequence revealed that the two obtained pegivirus strains were grouped in the *Pegivirus* *J* clade, which consists of rodent pegiviruses (Fig. 1B). The two obtained pegivirus strains were closest to the rat pegivirus RtRp-PegV/Cs2008 (DDBJ accession number MT085182) from a *Rattus* sp. in Cambodia (9), with nucleotide identities of 87.3% but showing only 59.1% to 59.3% identity with RPgV-CC61 (DDBJ accession number KC815311) from Neotoma lepida in the United States (10) within the near-entire coding region sequences.

In conclusion, we report the complete nucleotide sequences of two *Pegivirus J* strains recovered from *R. rattus* in Indonesia. The sequences determined in this study will be useful for further molecular virological and epidemiological studies of pegiviruses.

### Data availability.

The genome sequences described in this study have been deposited in DDBJ/EMBL/GenBank under accession numbers LC602140 and LC602141.
